# Volume of interest delineation techniques for ^18^F-FDG PET-CT scans during neoadjuvant extremity soft tissue sarcoma treatment in adults: a feasibility study

**DOI:** 10.1186/s13550-018-0397-1

**Published:** 2018-06-07

**Authors:** Marc G. Stevenson, Lukas B. Been, Harald J. Hoekstra, Albert J. H. Suurmeijer, Ronald Boellaard, Adrienne H. Brouwers

**Affiliations:** 1 Department of Surgical Oncology, University Medical Center Groningen, University of Groningen, Groningen, The Netherlands; 2 Department of Pathology and Medical Biology, University Medical Center Groningen, University of Groningen, Groningen, The Netherlands; 3 Department of Nuclear Medicine and Molecular Imaging, University Medical Center Groningen, University of Groningen, Hanzeplein 1, PO Box 30.001, 9700 RB Groningen, The Netherlands; 4 Department of Radiology and Nuclear Medicine, VU University Medical Center, Amsterdam, The Netherlands

**Keywords:** Soft tissue sarcoma, ^18^F-FDG PET-CT, Limb perfusion, Preoperative radiotherapy

## Abstract

**Background:**

This study explores various volume of interest (VOI) delineation techniques for fluorine-18-fluorodeoxyglucose positron emission tomography with computed tomography (^18^F-FDG PET-CT) scans during neoadjuvant extremity soft tissue sarcoma (ESTS) treatment.

**Results:**

During neoadjuvant treatment, hyperthermic isolated limb perfusion (HILP) and preoperative external beam radiotherapy (EBRT), 11 patients underwent three ^18^F-FDG PET-CT scans. The first scan was made prior to the HILP, the second after the HILP but prior to the start of the EBRT, and the third prior to surgical resection. An automatically drawn VOI_auto_, a manually drawn VOI_man_, and two gradient-based semi-automatically drawn VOIs (VOI_grad_ and VOI_grad+_) were obtained. Maximum standardized uptake value (SUV_max_), SUV_peak_, SUV_mean_, metabolically active tumor volume (MATV), and total lesion glycolysis (TLG) were calculated from each VOI. The correlation and level of agreement between VOI delineation techniques was explored. Lastly, the changes in metabolic tumor activity were related to the histopathologic response. The strongest correlation and an acceptable level of agreement was found between the VOI_man_ and the VOI_grad+_ delineation techniques. A decline (VOI_man_) in SUVmax, SUVpeak, SUVmean, TLG, and MATV (all *p* < 0.05) was found between the three scans. A > 75% decline in TLG between scan 1 and scan 3 possibly identifies histopathologic response.

**Conclusions:**

The VOI_grad+_ delineation technique was identified as most reliable considering reproducibility when compared with the other VOI delineation techniques during the multimodality neoadjuvant treatment of locally advanced ESTS. A significant decline in metabolic tumor activity during the treatment was found. TLG deserves further exploration as predictor for histopathologic response after multimodality ESTS treatment.

**Electronic supplementary material:**

The online version of this article (10.1186/s13550-018-0397-1) contains supplementary material, which is available to authorized users.

## Background

Soft tissue sarcomas (STS) are relatively rare malignancies, accounting for less than 1% of all cancers in adults. The number of patients presenting with STS each year is 600–700 in the Netherlands, leading to approximately 300 STS related deaths annually [1, 2].

Roughly 50–60% of the STS arise in the extremities [3, 4]. At presentation, some of these extremity soft tissue sarcomas (ESTS) are considered non-resectable or “locally advanced.” Since the 1990s, neoadjuvant hyperthermic isolated limb perfusion (HILP) has been used in Europe to prevent limb amputation in these patients [5], resulting in a limb salvage rate of 80–90% in locally advanced ESTS nowadays [6–9]. HILP is used in all types of adult locally advanced ESTS. It allows to administer regional chemotherapy in high doses, as the affected limb is isolated from the systemic circulation during the procedure. Neoadjuvant systemic chemotherapy in ESTS is currently under ongoing investigation, as the data available considering patients’ oncological outcome are inconsistent [10–12].

Fluorine-18-fluorodeoxyglucose positron emission tomography with computed tomography (^18^F-FDG PET-CT) scans have been used to evaluate tumor changes following HILP in locally advanced ESTS since the mid-1990s [13]. Pretreatment maximum standardized uptake value (SUV_max_), metabolically active tumor volume (MATV), and total lesion glycolysis (TLG) were identified as significant predictors for overall survival in STS in a recent meta-analysis [14]. Furthermore, post-treatment SUV_max_ was shown to be promising in monitoring treatment response. However, the identification of this latter parameter was solely based on two articles included in this meta-analysis. The first only included rhabdomyosarcomas, which is a chemosensitive sarcoma, and the second only included chest wall sarcomas [14–16].

The SUV_max_ of a lesion depends solely on the highest measured ^18^F-FDG uptake in one voxel, thereby making the measured SUV_max_ susceptible for noise [17]. Furthermore, the question remains whether this one measurement is representative for large, heterogeneous tumors, as STS. In contrast, the SUV_max_ is the most robust parameter when comparing various software delineation programs, delineation methods, and observers [18]. The outcome of MATV and TLG parameters are much more dependent of the method of tumor delineation and the software program used for these analyses. We hypothesized that the use of peak standardized uptake value (SUV_peak_) and mean standardized uptake value (SUV_mean_) in addition to SUV_max_, TLG, and MATV might result in a more reliable prediction of tumor changes induced by neoadjuvant treatment.

To the best of our knowledge, the use of various VOI delineation techniques has not yet been explored in and during the neoadjuvant treatment of STS. Furthermore, in this patient population, no sequential analysis of multiple ^18^F-FDG PET-CT scans has been performed previously. In this feasibility study, consecutive ^18^F-FDG PET-CT scans per patient were used to investigate the use of four VOI delineation techniques because variations in VOI will directly affect the measured SUV_mean_, MATV, and TLG and could thus affect the performance of the PET assessments. Furthermore, we explored the changes in metabolic tumor activity (SUV_max_, SUV_peak_, SUV_mean_, MATV, and TLG) to neoadjuvant HILP and preoperative EBRT during the treatment course of locally advanced ESTS. Lastly, the relationship between changes in metabolic tumor activity and histopathologic response was explored.

## Methods

**Fig. 1:**
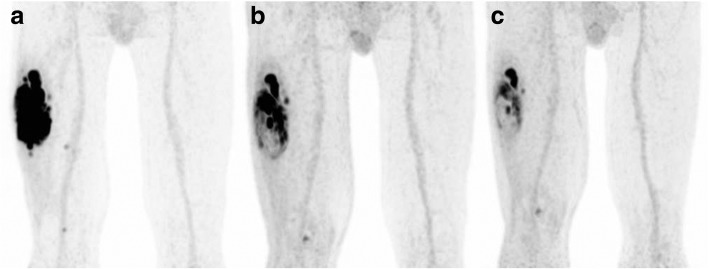
^18^F-FDG uptake throughout the tumor for one of the patients during the treatment course. Coronal ^18^F-FDG PET-CT images showing the heterogeneous ^18^F-FDG uptake throughout the tumor for one of the patients during the treatment course. **a** Scan 1 (baseline). **b** Scan 2 (after HILP). **c** Scan 3 (after EBRT)

This study has been approved by the Institutional Review Board (IRB), and the need for written informed consent was waived (IRB case number 2016.984). From 2011 to 2017, 11 patients with a median age of 64 (IQR 44–74; range 32–74) years were treated according to a novel treatment regimen consisting of neoadjuvant HILP, preoperative hypofractionated EBRT, followed by surgical resection of the tumor. All patients were diagnosed with a locally advanced, non-metastatic, high-grade ESTS (Table 1). Patients eligible for HILP treatment were included in this novel treatment regimen based on a tumor board decision. Inclusion and exclusion criteria, as well as treatment details, have been described in more detail elsewhere [19]. Patients were scheduled for three ^18^F-FDG PET-CT scans. The first scan was made prior to the start of neoadjuvant treatment (baseline) and the second after the HILP, but prior to the start of the preoperative EBRT and was additionally used for EBRT delineation. The third scan was made after completion of the neoadjuvant treatment (HILP and EBRT), but prior to surgical resection. Figure 1 illustrates the change in ^18^F-FDG uptake during the treatment course for one of the patients.

**Table 1:** Patient and tumor characteristics

Patient number	Gender	Age (years)	Histopathologic findings	Tumor location	Tumor size (cm)
1	M	32	Synovial sarcoma	Upper leg	6
2	F	41	Synovial sarcoma	Lower leg	4
3	F	74	Pleomorphic undifferentiated sarcoma	Upper leg	10
4	M	54	Pleomorphic undifferentiated sarcoma	Upper leg	17
5	M	63	Pleomorphic undifferentiated sarcoma	Lower leg	9
6	M	71	Myxofibrosarcoma	Upper leg	5
7	M	44	Myxofibrosarcoma	Upper leg	17
8	M	74	Pleomorphic undifferentiated sarcoma	Knee	7
9	M	64	Leiomyosarcoma	Knee	6
10	M	75	Pleomorphic undifferentiated sarcoma	Lower leg	8
11	M	67	Leiomyosarcoma	Knee	6

### ^18^F-FDG PET-CT

The ^18^F-FDG PET-CT scans were performed using a hybrid PET-CT scanner (Siemens Biograph mCT). Patients fasted at least 6 h prior to scanning, and fasting glucose levels were checked at time of injection; none of the patients suffered from diabetes mellitus. ^18^F-FDG (3 MBq/kg) was injected, and the PET-CT scan was started 1 h afterwards. Patients were scanned in supine position, and images of the affected limb were acquired in 3D mode, in two to five bed positions, 1–3 min/bed position based on the patient’s body weight. A preceding low dose CT scan was performed and used for attenuation and scatter correction. All images were reconstructed using an EARL compliant protocol; from 2011 to 2014, the images were reconstructed using the following reconstruction: 3i_24s, image size 400, filter Gaussian, and FWHM 5.0 mm, and from 2014 to 2017, the images were reconstructed with the following reconstruction parameters: 3i_21s, image size 256, filter Gaussian, FWHM 6.5 mm, and quality ref. mAS 30. All scans were acquired according to European Association of Nuclear Medicine guidelines (version 1.0/2.0) [20, 21].

### Image analyses

**Fig. 2:**
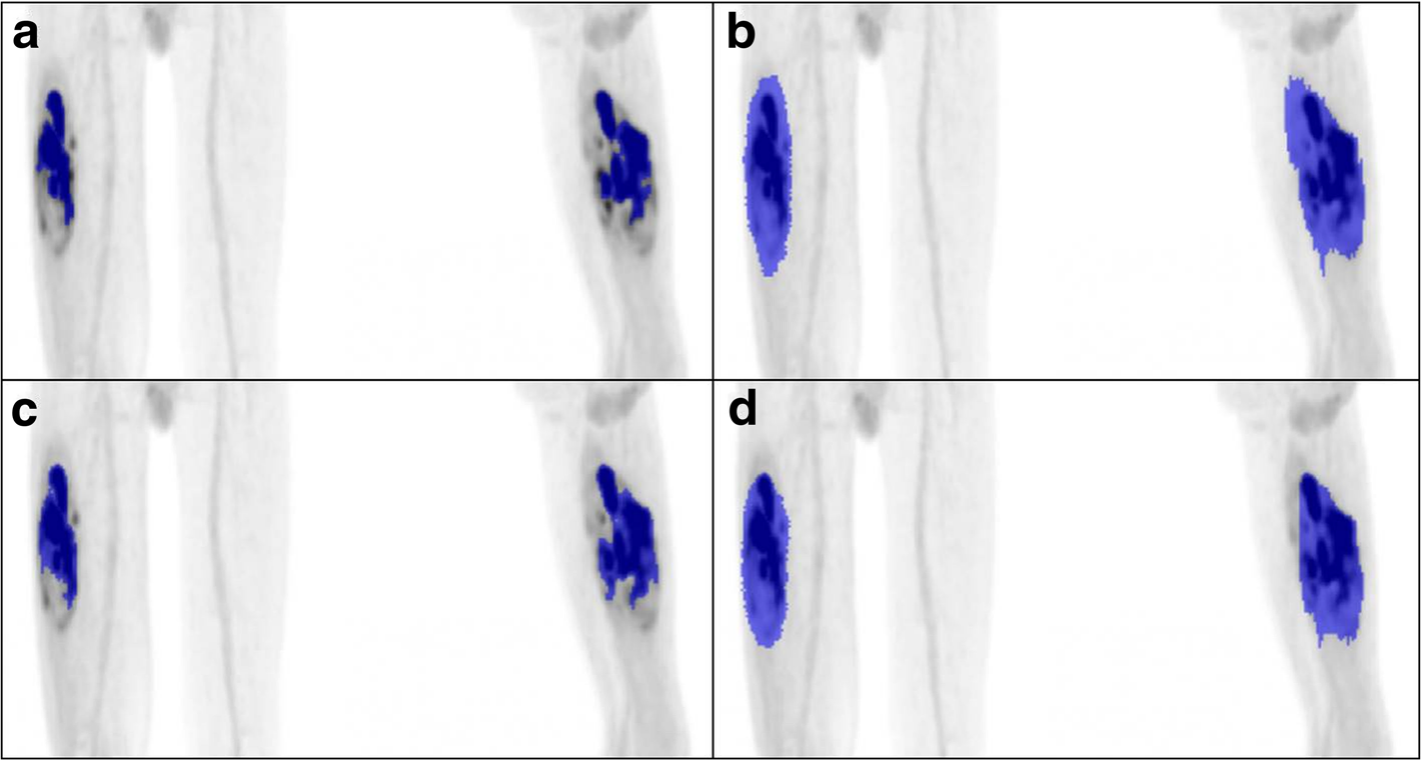
Differences in tumor delineation between the four VOI delineation techniques. An example illustrating the differences in tumor delineation between the four VOI delineation techniques, for patient 4 scan 2. **a** VOI_auto_. **b** VOI_man_. **c** VOI_grad_. **d** VOI_grad+_

Scans were imported into Accurate (in-house developed analysis software, as previously used by Frings and Kramer et al. [22, 23]) and recently described by Boellaard [24]. Scans were reviewed and analyzed by one researcher. To explore the effect of various delineation techniques on the measurement of the metabolic parameters, the volume of interest (VOI) of each tumor was drawn in four different ways: (1) an automatically drawn VOI_auto_ (using 50% of the SUV_peak_ contour, corrected for local background [22]), (2) a manually drawn VOI_man_ (visually following tumor contours), and (3) a semi-automatic drawn VOI_grad_ (a contour that is located at the maximum PET image intensity gradient near the boundary of the tumor). Because of tumor heterogeneity, necrotic tumor parts (mostly tumor centers) were not included in this third VOI. Therefore, a fourth VOI was derived from the VOI_grad_, in which all necrotic tumor parts were manually filled and included, resulting in the fourth VOI_grad+_ (Fig. 2).

Five metabolic parameters, SUV_max_ (voxel with the highest SUV value), SUV_peak_ (using a 1 mL sphere), SUV_mean_, TLG (SUV_mean_ × MATV), and MATV, all based on lean body mass, as recommended by Boellaard et al. [21], were derived for the four VOI delineation techniques.

Due to tumor necrosis in most tumors, either treatment-induced or due to tumor heterogeneity, only the VOI_man_ comprised the entire tumor (including necrosis). Therefore, the VOI_man_ was chosen as reference measurement, and the other VOI techniques were compared with the VOI_man_. We selected VOI_man_ as reference VOI for pragmatic reasons (as the VOI_man_ encompasses the entire tumor), not suggesting that this approach is best.

Correlation analyses, Bland-Altman analyses, and patient ranking were performed to compare correlation and level of agreement between the VOI delineation techniques. Bland-Altman analyses [25] and patient ranking are described in more detail in Additional file 1. Changes in metabolic tumor activity during neoadjuvant treatment were measured using the five metabolic parameters obtained from the reference VOI_man_ and were related to histopathologic responses. Histopathologic tumor responses were established in accordance with the European Organization for Research and Treatment of Cancer-Soft Tissue and Bone Sarcoma Group (EORTC-STBSG) STS response score [19]. Grade A represents no stainable tumor cells, grade B single stainable tumor cells or small clusters (overall below 1% of the whole specimen), grade C ≥ 1 to < 10% stainable tumor cells, grade D ≥ 10 to < 50% stainable tumor cells, and grade E ≥ 50% stainable tumor cells [26].

Histopathologic responders had tumor remnants which showed < 10% stainable cells, combining response grades A, B, and C. Non-responders had ≥ 10% stainable cells in their tumor remnant, grade D or E. Lastly, the relationship between changes in metabolic tumor activity and histopathologic responses was explored.

### Statistical analysis

Discrete variables were summarized with frequencies and percentages and continuous variables with medians and interquartile ranges (IQRs); none of the variables were normally distributed. Fisher’s exact and Mann-Whitney *U* test were used to compare variables. Wilcoxon signed rank and Friedman’s test were used to compare the measurements between the three scans. Correlation coefficients were calculated and tested using Spearman’s test. The level of agreement between VOI techniques was determined by Bland-Altman analyses [25]. A *p* value < 0.05 indicated statistical significance. Microsoft Excel (2010) was used to create the Bland-Altman plots. SPSS version 23.0 (IBM SPSS Statistics for Windows, Version 23.0 Armonk, NY: IBM Corp) and GraphPad Prism version 5.04 (GraphPad Software for Windows, San Diego California USA) were used for statistical analyses.

## Results

Thirty-two ^18^F-FDG PET-CT scans were acquired. The third PET-CT scan of patient 10 could not be performed due to scheduling difficulties. For patient 1, in scan 3 it was not possible to draw a VOI_auto_, since the tumor showed an almost complete metabolic response at this treatment stage and it did not meet the margin thresholds to complete the VOI_auto_. Since it was possible to define the other three types of VOIs, this scan was included in the analyses and a value of zero was given to the metabolic parameters for the VOI_auto_. The median time between the HILP and scan 2 was 21 [18–21] days, whereas the time between the end of EBRT and scan 3 was 3 (1–3) days.

### Correlation, level of agreement, and ranking of patients between VOIs

The correlation between VOIs for all scans and all metabolic parameters was strongest between the VOI_man_ and the VOI_grad+_, as indicated in gray in Table 2. The Bland-Altman plots showed an acceptable level of agreement between the VOI_man_ and the VOI_grad+_ (Additional file 2: Figure S1).

**Table 2:** Spearman’s correlation between the VOI_man_ and VOI_auto/grad/grad+_ for the serial ^18^F-FDG PET-CT scans

Parameter	Scan 1		Scan 2		Scan 3	
	Correlation coefficient	*p* value	Correlation coefficient	*p* value	Correlation coefficient	*p* value
SUV_max_						
VOI_man-auto_	1.000	NA	1.000	NA	0.988	< 0.001
VOI_man-grad_	1.000	NA	1.000	NA	1.000	NA
VOI_man-grad+_	1.000	NA	1.000	NA	1.000	NA
SUV_peak_						
VOI_man-auto_	1.000	NA	1.000	NA	0.988	< 0.001
VOI_man-grad_	1.000	NA	1.000	NA	1.000	NA
VOI_man-grad+_	1.000	NA	1.000	NA	1.000	NA
SUV_mean_						
VOI_man-auto_	0.964	< 0.001	0.836	0.001	0.564	0.090
VOI_man-grad_	0.991	< 0.001	0.882	< 0.001	0.758	0.011
VOI_man-grad+_	0.991	< 0.001	0.982	< 0.001	0.988	< 0.001
TLG						
VOI_man-auto_	0.845	0.001	0.982	< 0.001	0.842	0.002
VOI_man-grad_	0.991	< 0.001	1.000	NA	0.976	< 0.001
VOI_man-grad+_	0.991	< 0.001	0.991	< 0.001	0.988	< 0.001
MATV						
VOI_man-auto_	0.309	0.355	0.555	0.077	0.430	0.214
VOI_man-grad_	0.955	< 0.001	0.973	< 0.001	0.806	0.005
VOI_man-grad+_	0.936	< 0.001	1.000	NA	0.964	< 0.001

Spearman’s test for correlations was used to calculate significance. The strongest correlation for the three PET scans was found between the VOI_man_ and the VOI_grad+_, as indicated in gray

*VOI* volume of interest, *VOI*_*man*_ manually drawn VOI, *VOI*_*auto*_ automatically drawn VOI, *VOI*_*grad*_ VOI based on the gradient between voxels, *VOI*_*grad+*_ VOI_grad_ + necrosis, ^*18*^*F-FDG PET-CT* fluorine-18-fluorodeoxyglucose positron emission tomography with computed tomography, *SUV*_*max*_ maximum standardized uptake value, *SUV*_*peak*_ peak standardized uptake value, *SUV*_*mean*_ mean standardized uptake value, *TLG* total lesion glycolysis, *MATV* metabolically active tumor volume, *IQR* interquartile range, *NA* not applicable

No larger difference than 1 place in ranking for SUV_mean_, and TLG for the serial ^18^F-FDG PET-CT scans was found when comparing the VOI_man_ and the VOI_grad+_ delineation techniques, for the MATV no larger difference than 2 places in ranking was found. A relative large difference of 4 or more in ranking between VOI delineation techniques is indicated in gray in Additional file 3: Table S1. Among others, this was found for the MATV at scan 1 of patient 7 with considerable necrotic tumor parts. The measured MATV was found to be highest when using the VOI_man, grad and grad+_ techniques. However, when the VOI_auto_ technique was used, it was only ranked a 9th place due to exclusion of tumor necrosis.

### Metabolic tumor activity

**Fig. 3:**
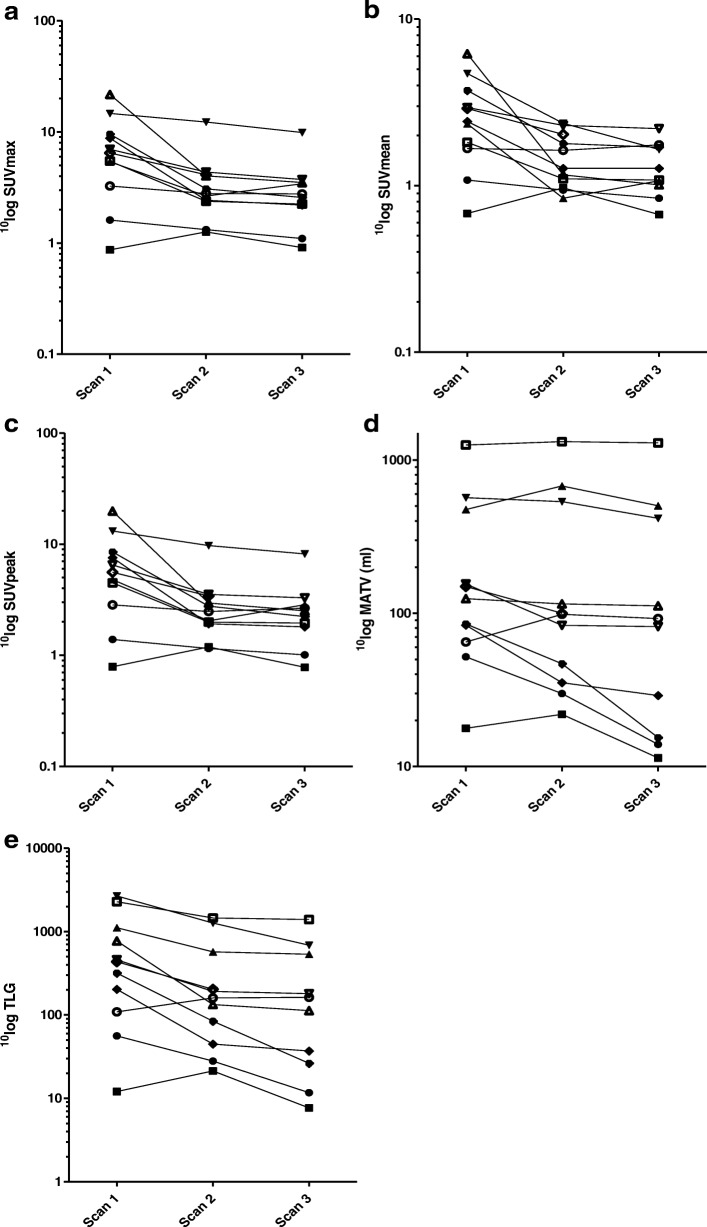
Course in metabolic tumor activity (VOI_man_) during neoadjuvant treatment for each patient individually. The course in metabolic tumor activity for the VOI_man_ during the neoadjuvant treatment for each patient individually for the serial ^18^F-FDG PET-CT scans. **a** SUV_max_. **b** SUV_mean_. **c** SUV_peak_. **d** Metabolically active tumor-volume (MATV). **e** Total lesion glycolysis (TLG)

During neoadjuvant treatment, all five metabolic parameters for the reference VOI_man_ declined between scans 1 and 3 (all *p* < 0.05, Fig. 3, Table 3).

**Table 3:** Metabolic tumor activity for the VOI_man_ for the serial ^18^F-FDG PET-CT scans

Parameter	Scan 1	Scan 2	Scan 3	*p* value
SUV_max_	6.5 (3.3–9.5)	2.8 (2.4–4.1)	2.7 (1.9–3.6)	0.002
SUV_peak_	5.6 (2.8–8.5)	2.5 (1.9–3.4)	2.4 (1.6–3.0)	0.001
SUV_mean_	2.4 (1.7–3.7)	1.3 (1.0–2.0)	1.2 (1.0–1.7)	0.006
TLG	434.8 (108.6–1112.8)	159.9 (44.7–570.9)	137.5 (22.6–572.6)	0.003
MATV (ml)	124.4 (64.8–474.2)	98.3 (35.2–534.2)	87.1 (15.1–437.7)	0.025

Data presented as median (IQR)

*VOI* volume of interest, *VOI*_*man*_ manually drawn VOI, ^*18*^*F-FDG PET-CT* fluorine-18-fluorodeoxyglucose positron emission tomography with computed tomography, *SUV*_*max*_ maximum standardized uptake value, *SUV*_*peak*_ peak standardized uptake value, *SUV*_*mean*_ mean standardized uptake value, *TLG* total lesion glycolysis, *MATV* metabolically active tumor volume, *IQR* interquartile range

**Fig. 4:**
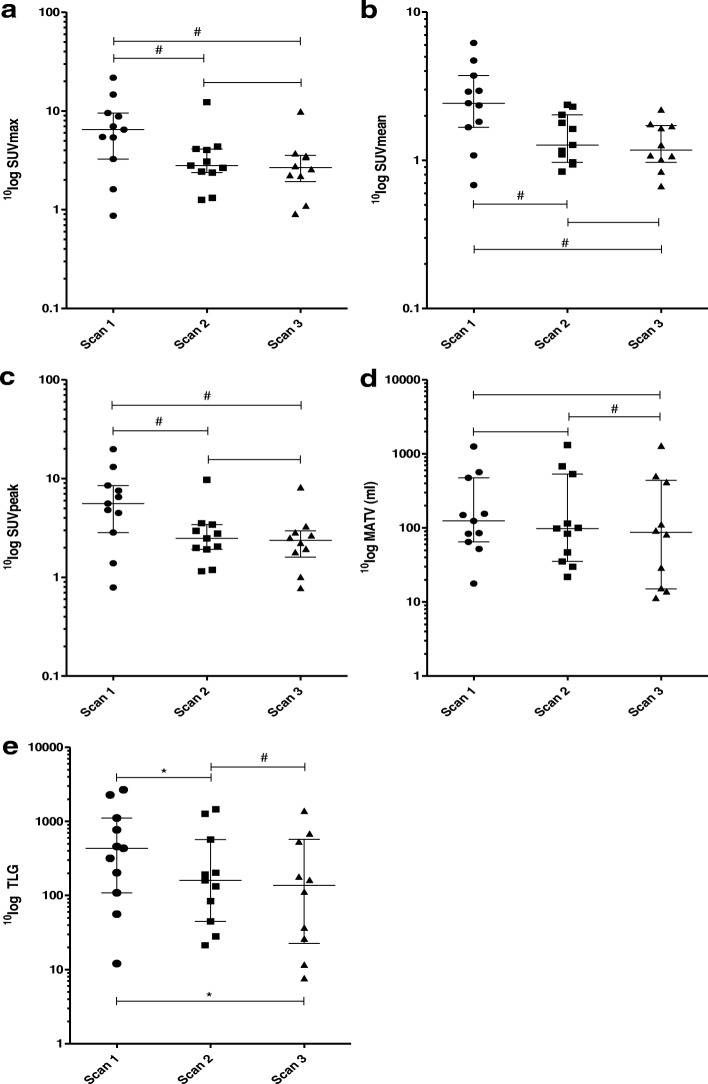
Changes in metabolic tumor activity (VOI_man_) during neoadjuvant treatment for the serial ^18^F-FDG PET-CT scans. Changes in metabolic tumor activity for the VOI_man_ during the neoadjuvant treatment for the serial ^18^F-FDG PET-CT scans. Median and interquartile ranges are indicated. **a** SUV_max_. **b** SUV_mean_. **c** SUV_peak_. **d** Metabolically active tumor volume (MATV). **e** Total lesion glycolysis (TLG). *p < 0.05; ^#^*p* < 0.01

This decline was further explored by calculating the absolute and the percentage difference between the three serial scans. The percentage difference was obtained by dividing the difference between scans by the measured value of the first scan. A significant decline in SUV_max_, SUV_peak_, and SUV_mean_ was found between scan 1 vs. scan 2, as well as between scan 1 vs. scan 3. However, no significant decline in SUV_max_, SUV_peak_, and SUV_mean_ was found between scan 2 vs. scan 3. The decline in TLG was significant between all serial scans. A significant decline in MATV was found between scan 2 vs. scan 3. The decline in metabolic tumor activity for all parameters except MATV was largest between scan 1 vs. 2, whereas the decline in MATV was largest between scan 2 vs. 3 (Fig. 4, Table 4).

**Table 4:** Changes in metabolic tumor activity for the VOI_man_ during the neoadjuvant treatment between the serial ^18^F-FDG PET-CT scans

Parameter	Scan 1 vs. 2		Scan 2 vs. 3		Scan 1 vs. 3	
	Δ	Δ %	Δ	Δ %	Δ	Δ %
SUV_max_	− 2.6 (− 6.4 to − 0.5)^#^	− 37.7 (− 67.7 to − 16.4)	− 0.3 (− 0.5 to − 0.1)	− 13.6 (− 17.5 to − 4.7)	− 3.2 (− 6.7 to − 0.5)^#^	− 41.6 (− 73.5 to − 27.7)
SUV_peak_	−2.8 (− 5.6 to − 0.4)^#^	− 45.8 (− 67.4 to − 17.3)	− 0.2 (− 0.5 to 0.0)	− 9.3 (− 16.8 to 0.4)	− 2.9 (− 5.9 to − 0.3)^#^	− 45.1 (− 74.4 to − 21.9)
SUV_mean_	− 0.9 (− 1.9 to − 0.1)^#^	− 39.3 (− 52.0 to − 13.2)	− 0.1 (− 0.2 to 0.0)	− 4.6 (− 17.2 to 2.3)	− 1.0 (− 2.3 to − 0.2)^#^	− 44.1 (− 57.2 to − 17.0)
TLG	− 233.6 (− 637.9 to − 28.0)*	− 52.6 (− 73.6 to − 36.3)	− 18.4 (− 57.0 to − 10.6)^#^	− 16.3 (− 59.7 to − 5.5)	− 285.0 (− 714.7 to − 34.4)*	− 67.5 (− 82.6 to − 38.2)
MATV (ml)	− 22.1 (− 48.2 to 33.5)	− 7.5 (− 44.9 to 23.5)	− 13.2 (− 53.0 to − 5.3)^#^	− 19.8 (− 49.4 to − 2.7)	− 25.4 (− 70.6 to 27.6)	− 31.2 (− 67.2 to 3.8)

Data presented as median (IQR)

*VOI* volume of interest, *VOI*_*man*_ manually drawn VOI, ^*18*^*F-FDG PET-CT* fluorine-18-fluorodeoxyglucose positron emission tomography with computed tomography, *SUV*_*max*_ maximum standardized uptake value, *SUV*_*peak*_ peak standardized uptake value, *SUV*_*mean*_ mean standardized uptake value, *TLG* total lesion glycolysis, *MATV* metabolically active tumor volume, *IQR* interquartile range. *p < 0.05; ^#^*p* < 0.01

### Histopathologic response

Histopathologic response to neoadjuvant treatment varied among the 11 patients, as follows: one grade A (9.1%), one grade B (9.1%), two grade C (18.2%) (totaling to four histopathologic responders (36.4%)), five grade D (45.5%), and two grade E (18.2%) (totaling to 7 non-responders (64.4%)). The histopathologic responders seem to be identifiable by a decline in TLG of > 75% between scans 1 and 3 calculated using the VOI_man_ (Table 5).

**Table 5:** Changes in metabolic tumor activity for the VOI_man_ during the neoadjuvant treatment between ^18^F-FDG PET-CT scans 1 and 3, combined with the corresponding histopathologic tumor response for each patient

Patient number	Scan 1 vs. 3 SUV_max_		Scan 1 vs. 3 SUV_peak_		Scan 1 vs. 3 SUV_mean_		Scan 1 vs. 3 TLG		Scan 1 vs. 3 MATV (ml)		EORTC-STBSG response grade [26]
	Δ	Δ %	Δ	Δ %	Δ	Δ %	Δ	Δ %	Δ	Δ %	
1	− 0.5	− 32.0	− 0.4	− 27.0	− 0.2	− 22.4	− 44.4	− 79.2	− 38.0	− 73.1	C
2	0.1	5.2	− 0.0	− 1.7	0.0	− 0.7	− 4.4	− 36.3	− 6.4	− 35.8	D
3	− 2.0	− 36.6	− 2.0	− 40.7	− 1.3	− 54.6	− 578.1	− 51.9	27.9	5.9	D
4	− 4.8	− 32.7	− 4.9	− 37.6	− 3.1	− 65.1	− 1986.4	− 74.3	− 150.4	− 26.5	D
5	− 6.6	− 75.0	− 5.7	− 76.2	− 1.2	− 47.6	− 165.7	− 81.8	− 54.4	− 65.2	A
6	− 0.5	− 15.0	− 0.2	− 6.4	0.1	5.0	53.8	49.5	27.5	42.4	E
7	− 3.2	− 59.0	− 2.5	− 56.5	− 0.7	− 40.6	− 883.9	− 38.8	38.6	3.1	D
8	− 18.2	− 83.8	− 17.3	− 87.3	− 5.2	− 83.7	− 658.4	− 85.4	− 12.8	− 10.3	B
9	− 3.2	− 46.5	− 3.2	− 49.5	− 0.8	− 25.5	− 278.7	− 60.7	− 73.5	− 47.3	D
10	NA	NA	NA	NA	NA	NA	NA	NA	NA	NA	E
11	− 7.0	− 73.1	− 6.3	− 73.8	− 2.0	− 54.4	− 291.3	− 91.7	− 69.6	− 81.9	C

Histopathologic responders are indicated in gray. A percentage difference of > 75% in TLG seemed to identify the histopathologic responders; these values were encircled

^*18*^*F-FDG PET-CT* fluorine-18-fluorodeoxyglucose positron emission tomography with computed tomography, *SUV*_*max*_ maximum standardized uptake value, *SUV*_*peak*_ peak standardized uptake value, *SUV*_*mean*_ mean standardized uptake value, *TLG* total lesion glycolysis, *MATV* metabolically active tumor volume, *EORTC-STBSG* European Organization for Research and Treatment of Cancer-Soft Tissue and Bone Sarcoma Group

To further explore the identification of the histopathologic responders, the difference and percentage difference in TLG between scans 1 and 3 for the four VOI delineation techniques was calculated (Additional file 4: Table S2). A calculated decline in TLG of > 75% using the VOI_grad/grad+_ identified the same histopathologic responders as the VOI_man_. The VOI_auto_ however failed to identify patient 5 as histopathologic responder. Furthermore, a > 75% decline in TLG was also found with the VOI_auto_ and VOI_grad_ in patients 3 and 4 and with the VOI_grad+_ in patient 4.

## Discussion

This study studying four VOI delineation techniques in three consecutive ^18^F-FDG PET-CT scans per patient demonstrates a significant decline in metabolic tumor activity (VOI_man_) during the neoadjuvant treatment, consisting of HILP and preoperative EBRT, of locally advanced ESTS. The decline in SUV_max_, SUV_peak_, SUV_mean_, and TLG between scan 1 vs. 2 implies that the HILP accounts for the largest effect on metabolic tumor activity. The MATV seems to be affected most by the EBRT, given the significant decline found between scan 2 vs. 3.

In search of a uniform and reproducible way to calculate changes in metabolic tumor activity in these upfront highly heterogeneous tumors, the use of four different VOI delineation techniques was studied. The VOI_man_ (defined as reference VOI) is the only delineation technique in which the entire tumor is encompassed independently of the amount of necrosis present in the tumor. Therefore, the VOI_man_ delineation technique seems to be most reliable when used for calculating the metabolic tumor activity. However, the VOI_man_ delineation technique is time-consuming, making it unfit for implementation into daily practice. A high correlation, acceptable level of agreement, and comparable ranking was found between the VOI_man_ and the VOI_grad+_ delineation techniques. The differences in ranking between the four VOI delineation techniques are best explained by the high amount of necrosis present in these tumors, as tumor necrosis did not meet the margin thresholds of the VOI_auto_ and VOI_grad_. To obtain the VOI_grad+_, the necrosis was manually included, and therefore, the ranking of patients was comparable to the ranking according to the VOI_man_.

Thus, the VOI_grad+_ delineation technique seems to be a reliable and reproducible technique for the delineation of heterogeneous tumors as ESTS. Further studies including larger patient cohorts in various solid tumor types are necessary for the validation and reproducibility of the various VOI delineation techniques. This study, however, demonstrates that the applied VOI delineation technique is important to consider because we found that assessment of response based on metabolic parameters derived from different VOIs may differ across subjects.

The metabolic tumor changes during neoadjuvant treatment between scan 1 vs. scan 3 were analyzed and compared with the corresponding histopathologic tumor response. Out of the five metabolic parameters tested, TLG seemed to identify the histopathologic responders most reliably (> 75% decrease in TLG between scan 1 and scan 3) when using the VOI_man_ delineation technique. Using the 75% decrease in TLG as a cutoff value was derived empirically from the data, used as example, and to obtain pilot data for using and comparing these techniques. When compared with the VOI_man_ delineation technique, the VOI_grad+_ technique identified the same histopathologic responders with only one additional patient. It seems that these two delineation techniques most reliably identify histopathologic responders, because they include tumor necrosis. The difference in performance of the VOI_man_ and VOI_grad+_ delineation techniques in identifying histopathologic responders is very subtle. However, the VOI_grad+_ delineation technique was found to be easier in use and is considerably less time-consuming than the VOI_man_ technique, making it more suitable for implementation into daily practice. The VOI delineation techniques and the TLG cutoff value need confirmation in larger patient cohorts.

During the last years, the predictive value of ^18^F-FDG PET-CT scans in staging and monitoring treatment response during neoadjuvant treatment has been established for various solid tumors (including metastatic colorectal cancer and non-small cell lung cancer [23, 27–29]. Therefore, further ESTS studies in which metabolic tumor activity, e.g., > 75% decrease in TLG with VOI_man_ and/or VOI_grad+_, is explored as predictor for monitoring therapy response, for histopathologic findings, and for oncological outcome are warranted. The identification of reproducible and reliable VOI delineation techniques, as well as the identification of robust PET parameters for the interpretation of changes in metabolic tumor activity, is relevant because this will enable clinicians to shorten delineation time and to compare results between observers, patients, and centers for ESTS and for other solid tumor types.

This study has some limitations, such as the retrospective character and the small patient population of the study. Only 11 patients were included; however, all patients but one underwent all three ^18^F-FDG PET-CT scans, and therefore, it was possible to establish the changes in metabolic tumor activity during the neoadjuvant treatment in all patients. Possibly, the interpretation of the third PET scan is biased by local inflammatory changes following the EBRT. These inflammatory changes might partly explain the significantly more pronounced decrease in metabolic tumor activity following the HILP then following the EBRT, as found in the current series. Despite this potential bias due to radiation-induced local inflammatory changes, a decrease in metabolic tumor activity between scans 1 and 3 was found, which theoretically might have been larger without these changes. For the purpose of this study, all data considering the metabolic tumor activity were obtained from an additional analyses of the ^18^F-FDG PET-CT scans, since these data are not used in routine patient care. Interestingly, the EORTC-STBSG response score [26] could be used to explore the relationship between changes in metabolic tumor activity and histopathologic response. However, the prognostic value of the STS response score according to the proportion of stainable tumor cells needs further validation [30].

## Conclusions

This study identified the VOI_grad+_ delineation technique as most reliable considering reproducibility when compared with the other delineation techniques during the multimodality neoadjuvant treatment of locally advanced ESTS. Moreover, the VOI_grad+_ delineation technique was considerably less time-consuming to perform when compared to the VOI_man_ technique, potentially resulting in easier implementation in clinical practice. A significant decline in metabolic tumor activity during the treatment was found. The decrease in metabolic tumor activity was significantly more pronounced after HILP than after preoperative radiotherapy. TLG seems promising, but warrants further confirmation, as predictor for histopathologic response in ESTS. Further studies in larger ESTS patient cohorts in which the investigated metabolic parameters and VOI delineation techniques are confirmed and validated as predictors for monitoring treatment response, for histopathologic response, and for oncological outcome are warranted, as this will result in an increase in the clinical applicability of metabolic tumor activity assessments in longitudinal sarcoma ^18^F-FDG PET-CT studies.

## Additional files

Additional file 1: Supplemental Methods. (DOCX 17 kb)

Additional file 2: **Figure S1.** Bland-Altman plots showing the level of agreement between the VOI_man_ and the VOI_auto/grad/grad+_ for the serial ^18^F-FDG PET-CT scans for A SUV_mean_, B total lesion glycolysis (TLG), and C metabolically active tumor-volume (MATV). (PDF 529 kb)

Additional file 3: **Table S1.** Ranking of patients for SUV_mean_, TLG, and MATV according to the four VOI delineation techniques. (DOCX 26 kb)

Additional file 4: **Table S2.** Changes in TLG according to the four VOI delineation techniques between ^18^F-FDG PET-CT scan 1 and scan 3, combined with the corresponding histopathologic tumor response for each patient. (DOCX 22 kb)

## Abbreviations

^18^F-FDG PET-CT: Fluorine-18-fluorodeoxyglucose positron emission tomography with computed tomography
EBRT: External beam radiotherapy
EORTC-STBSG: European Organization for Research and Treatment of Cancer-Soft Tissue and Bone Sarcoma Group
ESTS: Extremity soft tissue sarcoma
HILP: Hyperthermic isolated limb perfusion
IQR: Interquartile range
IRB: Institutional Review Board
MATV: Metabolically active tumor volume
STS: Soft tissue sarcoma
SUV_max_: Maximum standardized uptake value
SUV_mean_: Mean standardized uptake value
SUV_peak_: Peak standardized uptake value
TLG: Total lesion glycolysis
VOI: Volume of interest
