# Supplementary material for: Volume of interest delineation techniques for 18F-FDG PET-CT scans during neoadjuvant extremity soft tissue sarcoma treatment in adults: a feasibility study
Source: EJNMMI Res. 2018 Jun 7;8:42. doi: 10.1186/s13550-018-0397-1 (PMC5992109; doi:10.1186/s13550-018-0397-1)
Supplement: Supplementary file 1 — Supplemental Methods. (DOCX 17 kb) [file 13550_2018_397_MOESM1_ESM.docx]

Additional file 1

*Bland-Altman analyses*

Bland-Altman analyses were performed to determine the level of agreement between volume of interest (VOI) delineation techniques. Bland-Altman plots were created to compare the reference VOI_man_ with the other three VOI delineation techniques. Plots comparing the difference vs. the average as well as the percentage difference vs. the average between the VOI_man_ and the three other VOI delineation techniques were created. The percentage difference was obtained by dividing the difference between the measured values by the average of these values. This was performed for SUVmean, TLG and MATV, and not for SUVmax and SUVpeak, since the measured values for these latter parameters were identical for all scans, independently of the VOI delineation technique that was used.

*Ranking of patients*

Patients were ranked according to the SUVmean, TLG and MATV for each scan. SUVmax and SUVpeak were not included, for the same reason as stated above. The highest value was given rank 1 and the lowest value was given rank 11. Using this ranking method, the VOI delineation techniques were compared. A difference in ranking of four or more between the highest and lowest rank was indicated in gray.
