# Supplementary material for: Volume of interest delineation techniques for 18F-FDG PET-CT scans during neoadjuvant extremity soft tissue sarcoma treatment in adults: a feasibility study
Source: EJNMMI Res. 2018 Jun 7;8:42. doi: 10.1186/s13550-018-0397-1 (PMC5992109; doi:10.1186/s13550-018-0397-1)
Supplement: Supplementary file 2 — Figure S1. Bland-Altman plots showing the level of agreement between the VOIman and the VOIauto/grad/grad+ for the serial 18F-FDG PET-CT scans for A SUVmean, B total lesion glycolysis (TLG), and C metabolically active tumor-volume (MATV). (PDF 529 kb) [file 13550_2018_397_MOESM2_ESM.pdf]

Figure S1. Bland-Altman plots showing the level of agreement between the VOI_{man} and the $VOI_{auto/grad/grad+}$ for the serial ^{18}F-FDG PET-CT scans for: **A** SUVmean, **B** total lesion glycolysis (TLG), **C** metabolically active tumor-volume (MATV).

A

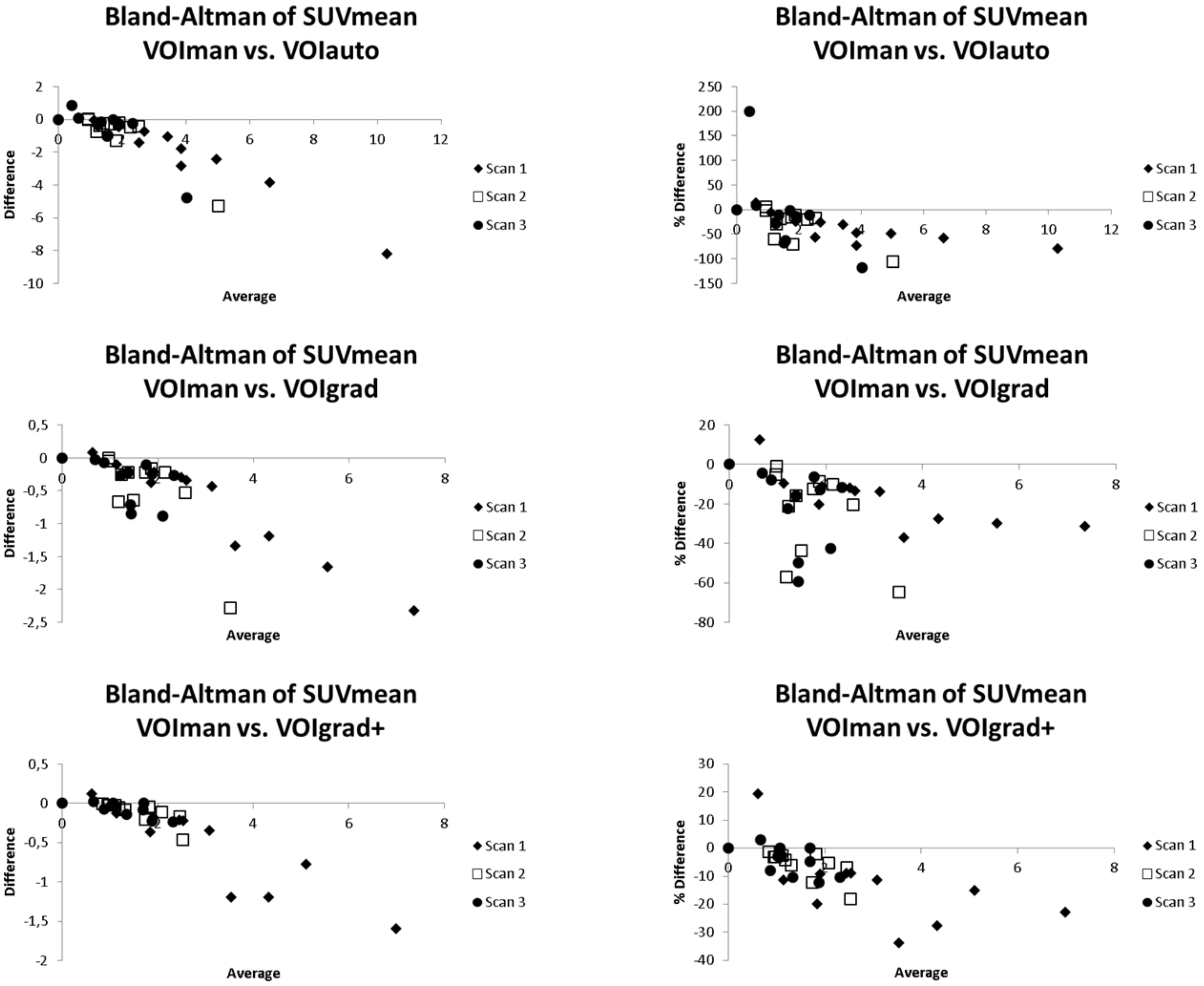

B

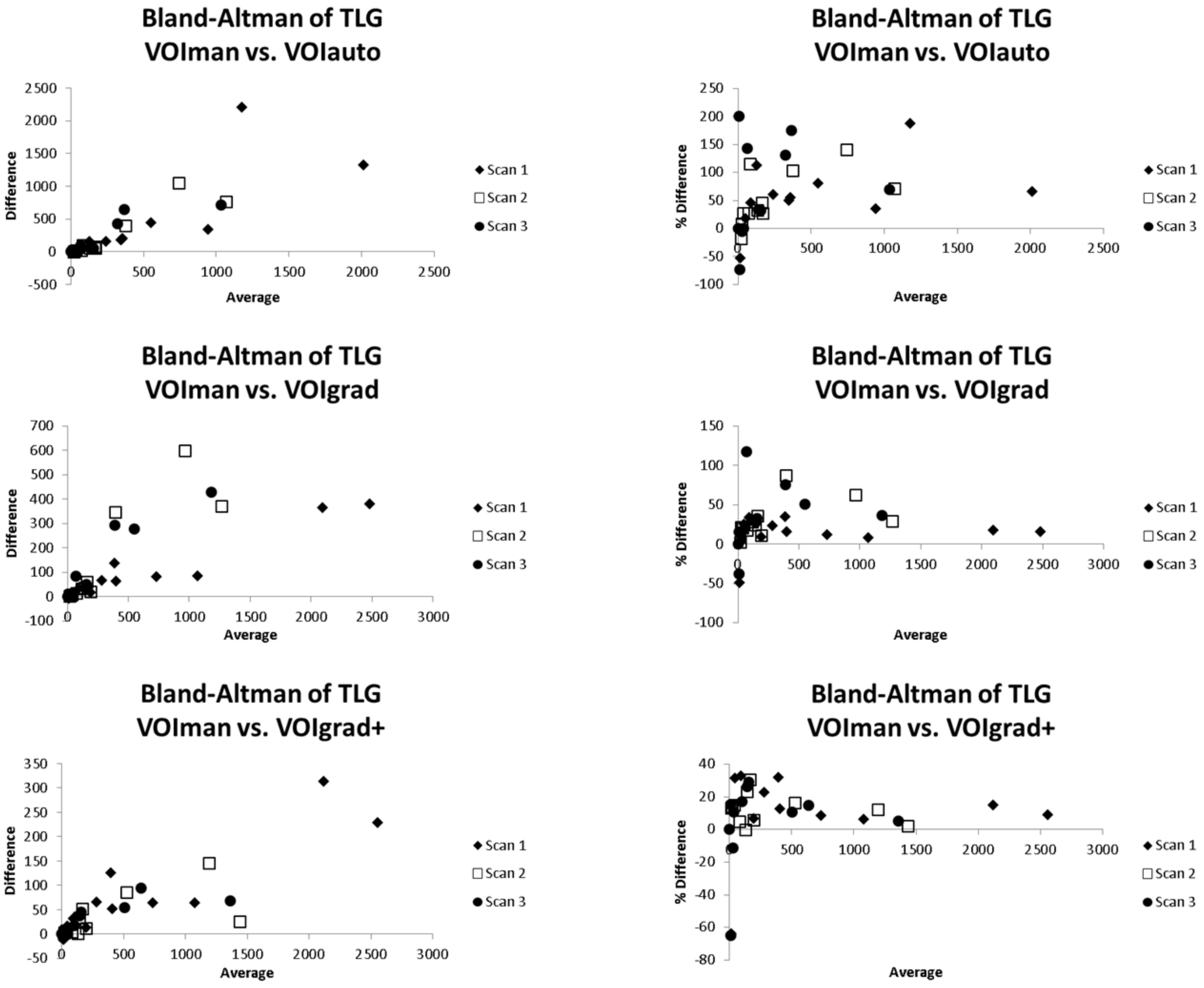
Bland-Altman of TLG
VOIman vs. VOIauto
Difference
Average
Scan 1
Scan 2
Scan 3
Bland-Altman of TLG
VOIman vs. VOIauto
% Difference
Average
Scan 1
Scan 2
Scan 3
Bland-Altman of TLG
VOIman vs. VOIgrad
Difference
Average
Scan 1
Scan 2
Scan 3
Bland-Altman of TLG
VOIman vs. VOIgrad
% Difference
Average
Scan 1
Scan 2
Scan 3
Bland-Altman of TLG
VOIman vs. VOIgrad+
Difference
Average
Scan 1
Scan 2
Scan 3
Bland-Altman of TLG
VOIman vs. VOIgrad+
% Difference
Average
Scan 1
Scan 2
Scan 3

C

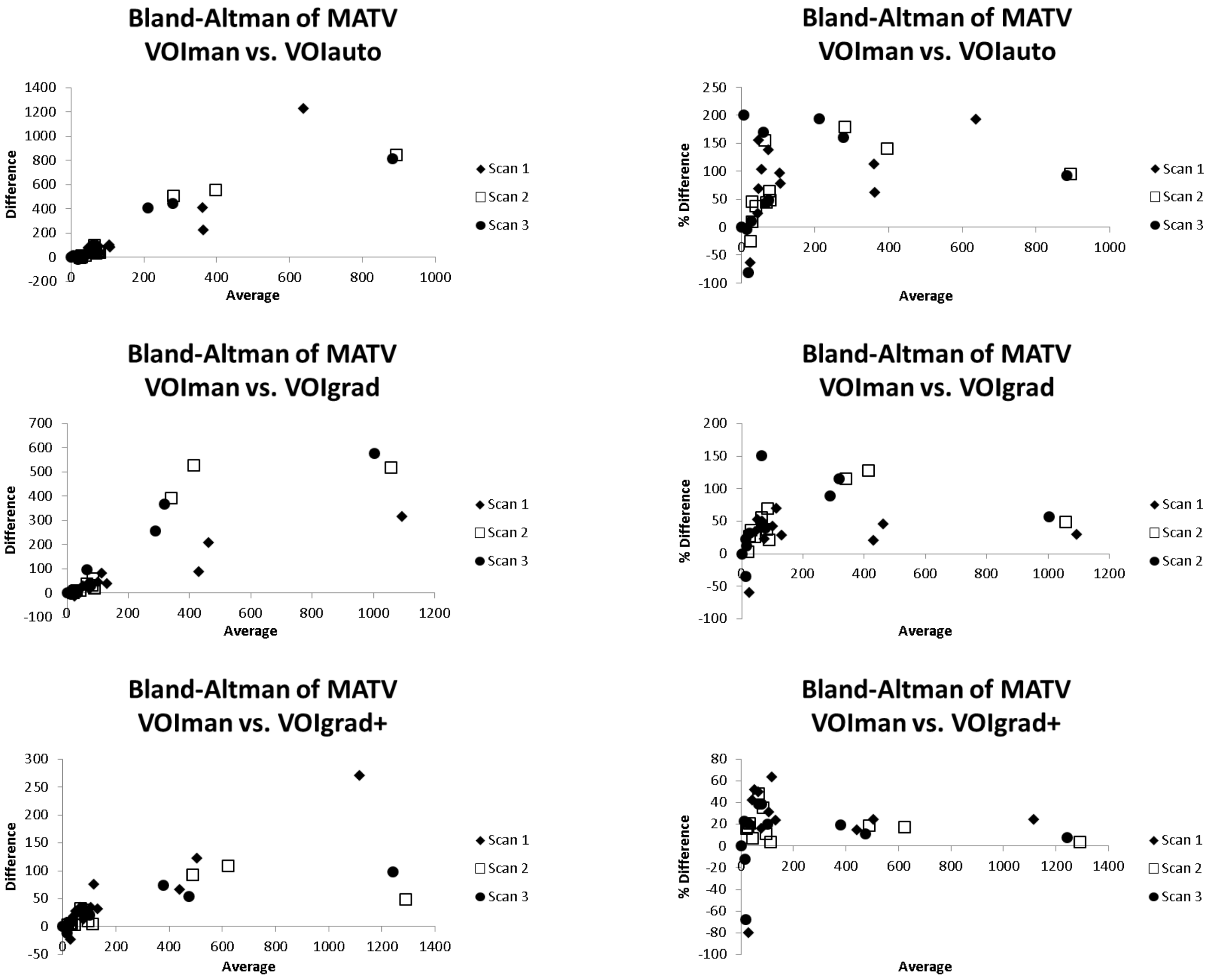

Bland-Altman of MATV VOIman vs. VOIauto
Difference
Average
Scan 1
Scan 2
Scan 3
Bland-Altman of MATV VOIman vs. VOIauto
% Difference
Average
Scan 1
Scan 2
Scan 3
Bland-Altman of MATV VOIman vs. VOIgrad
Difference
Average
Scan 1
Scan 2
Scan 3
Bland-Altman of MATV VOIman vs. VOIgrad
% Difference
Average
Scan 1
Scan 2
Scan 2
Bland-Altman of MATV VOIman vs. VOIgrad+
Difference
Average
Scan 1
Scan 2
Scan 3
Bland-Altman of MATV VOIman vs. VOIgrad+
% Difference
Average
Scan 1
Scan 2
Scan 3
