# Supplementary material for: Volume of interest delineation techniques for 18F-FDG PET-CT scans during neoadjuvant extremity soft tissue sarcoma treatment in adults: a feasibility study
Source: EJNMMI Res. 2018 Jun 7;8:42. doi: 10.1186/s13550-018-0397-1 (PMC5992109; doi:10.1186/s13550-018-0397-1)
Supplement: Supplementary file 3 — Table S1. Ranking of patients for SUVmean, TLG, and MATV according to the four VOI delineation techniques. (DOCX 26 kb) [file 13550_2018_397_MOESM3_ESM.docx]

Table S1: Ranking of patients for SUVmean, TLG and MATV according to the four VOI delineation techniques

| **Patient** | **SUVmean scan 1** | | | | **SUVmean scan 2** | | | | **SUVmean scan 3** | | | |
| --- | --- | --- | --- | --- | --- | --- | --- | --- | --- | --- | --- | --- |
| **number** | **VOI_man_** | **VOI_auto_** | **VOI_grad_** | **VOI_grad+_** | **VOI_man_** | **VOI_auto_** | **VOI_grad_** | **VOI_grad+_** | **VOI_man_** | **VOI_auto_** | **VOI_grad_** | **VOI_grad+_** |
| 1 | 10 | 10 | 10 | 10 | 10 | 10 | 10 | 10 | 9 | 10 | 9 | 9 |
| 2 | 11 | 11 | 11 | 11 | 9 | 11 | 11 | 9 | 10 | 9 | 10 | 10 |
| 3 | 7 | 8 | 7 | 7 | 11 | 7 | 7 | 11 | 7 | 3 | 6 | 7 |
| 4 | 2 | 2 | 2 | 2 | 1 | 1 | 1 | 2 | 4 | 1 | 1 | 3 |
| 5 | 6 | 4 | 6 | 6 | 6 | 8 | 8 | 4 | 5 | 8 | 7 | 5 |
| 6 | 9 | 9 | 8 | 8 | 5 | 6 | 5 | 6 | 2 | 4 | 3 | 2 |
| 7 | 8 | 7 | 9 | 9 | 8 | 9 | 9 | 8 | 6 | 7 | 8 | 6 |
| 8 | 1 | 1 | 1 | 1 | 7 | 4 | 6 | 7 | 8 | 5 | 4 | 8 |
| 9 | 4 | 5 | 4 | 4 | 2 | 2 | 2 | 1 | 1 | 2 | 2 | 1 |
| 10 | 5 | 6 | 5 | 5 | 3 | 3 | 3 | 3 | N/A | N/A | N/A | N/A |
| 11 | 3 | 3 | 3 | 3 | 4 | 5 | 4 | 5 | 3 | 6 | 5 | 4 |
| **Patient** | **TLG scan 1** | | | | **TLG scan 2** | | | | **TLG scan 3** | | | |
| **number** | **VOI_man_** | **VOI_auto_** | **VOI_grad_** | **VOI_grad+_** | **VOI_man_** | **VOI_auto_** | **VOI_grad_** | **VOI_grad+_** | **VOI_man_** | **VOI_auto_** | **VOI_grad_** | **VOI_grad+_** |
| 1 | 10 | 10 | 10 | 10 | 10 | 10 | 10 | 10 | 9 | 10 | 10 | 10 |
| 2 | 11 | 11 | 11 | 11 | 11 | 11 | 11 | 11 | 10 | 9 | 9 | 9 |
| 3 | 3 | 2 | 3 | 3 | 3 | 3 | 3 | 3 | 3 | 4 | 3 | 3 |
| 4 | 1 | 1 | 1 | 1 | 2 | 2 | 2 | 2 | 2 | 5 | 2 | 2 |
| 5 | 8 | 9 | 8 | 8 | 9 | 9 | 9 | 9 | 7 | 6 | 6 | 7 |
| 6 | 9 | 8 | 9 | 9 | 6 | 6 | 6 | 7 | 5 | 3 | 5 | 5 |
| 7 | 2 | 7 | 2 | 2 | 1 | 1 | 1 | 1 | 1 | 1 | 1 | 1 |
| 8 | 4 | 3 | 4 | 4 | 7 | 8 | 7 | 6 | 6 | 8 | 7 | 6 |
| 9 | 5 | 5 | 6 | 6 | 5 | 4 | 5 | 5 | 4 | 2 | 4 | 4 |
| 10 | 6 | 4 | 5 | 5 | 4 | 5 | 4 | 4 | N/A | N/A | N/A | N/A |
| 11 | 7 | 6 | 7 | 7 | 8 | 7 | 8 | 8 | 8 | 7 | 8 | 8 |
| **Patient** | **MATV scan 1** | | | | **MATV scan 2** | | | | **MATV scan 3** | | | |
| **number** | **VOI_man_** | **VOI_auto_** | **VOI_grad_** | **VOI_grad+_** | **VOI_man_** | **VOI_auto_** | **VOI_grad_** | **VOI_grad+_** | **VOI_man_** | **VOI_auto_** | **VOI_grad_** | **VOI_grad+_** |
| 1 | 10 | 5 | 10 | 11 | 10 | 9 | 10 | 10 | 9 | 10 | 10 | 10 |
| 2 | 11 | 6 | 11 | 9 | 11 | 8 | 11 | 11 | 10 | 5 | 7 | 8 |
| 3 | 3 | 1 | 2 | 3 | 2 | 2 | 2 | 2 | 2 | 3 | 3 | 2 |
| 4 | 2 | 2 | 3 | 2 | 3 | 7 | 3 | 3 | 3 | 9 | 2 | 3 |
| 5 | 8 | 11 | 7 | 7 | 9 | 10 | 9 | 9 | 7 | 6 | 6 | 7 |
| 6 | 9 | 7 | 9 | 10 | 6 | 3 | 5 | 6 | 5 | 2 | 4 | 5 |
| 7 | 1 | 9 | 1 | 1 | 1 | 1 | 1 | 1 | 1 | 1 | 1 | 1 |
| 8 | 6 | 10 | 5 | 5 | 4 | 11 | 6 | 4 | 4 | 8 | 8 | 4 |
| 9 | 4 | 4 | 6 | 6 | 7 | 4 | 7 | 7 | 6 | 4 | 5 | 6 |
| 10 | 5 | 3 | 4 | 4 | 5 | 5 | 4 | 5 | N/A | N/A | N/A | N/A |
| 11 | 7 | 8 | 8 | 8 | 8 | 6 | 8 | 8 | 8 | 7 | 9 | 9 |

Rank 1 is given for the highest value, and rank 11 for the lowest value calculated for SUVmean, TLG and MATV for all scans. In gray: a difference of four or more between the highest and lowest rank. Abbreviations: VOI= volume of interest; VOI_man_= manually drawn VOI; SUVmean= mean standardized uptake value; TLG= total lesion glycolysis; MATV= metabolically active tumor-volume; NA= not applicable
