# Supplementary material for: Volume of interest delineation techniques for 18F-FDG PET-CT scans during neoadjuvant extremity soft tissue sarcoma treatment in adults: a feasibility study
Source: EJNMMI Res. 2018 Jun 7;8:42. doi: 10.1186/s13550-018-0397-1 (PMC5992109; doi:10.1186/s13550-018-0397-1)
Supplement: Supplementary file 4 — Table S2. Changes in TLG according to the four VOI delineation techniques between 18F-FDG PET-CT scan 1 and scan 3, combined with the corresponding histopathologic tumor response for each patient. (DOCX 22 kb) [file 13550_2018_397_MOESM4_ESM.docx]

Table S2. Changes in TLG according to the four VOI delineation techniques between ^18^F-FDG PET-CT scan 1 and scan 3, combined with the corresponding histopathologic tumor response for each patient.

| Patient  number | VOI_man_  TLG | | VOI_auto_  TLG | | VOI_grad_  TLG | | VOI_grad+_  TLG | | EORTC-STBSG  response Grade*(21)* |
| --- | --- | --- | --- | --- | --- | --- | --- | --- | --- |
|  | **Δ** | **Δ %** | **Δ** | **Δ %** | **Δ** | **Δ %** | **Δ** | **Δ %** |  |
| 1 | -44.4 | -79.2 | -47.3 | -100.0 | -33.5 | -77.0 | -30.8 | -75.4 | C |
| 2 | -4.4 | -36.3 | -4.1 | -20.0 | -8.7 | -43.7 | -8.3 | -35.7 | D |
| 3 | -578.1 | -51.9 | -665.8 | -85.6 | -786.3 | -76.5 | -568.7 | -54.2 | D |
| 4 | -1986.4 | -74.3 | -1308.0 | -96.7 | -1886.6 | -82.3 | -1852.9 | -75.8 | D |
| 5 | -165.7 | -81.8 | -19.5 | -34.6 | -153.6 | -83.0 | -156.3 | -82.4 | A |
| 6 | 53.8 | 49.5 | 47.4 | 69.3 | 45.7 | 58.9 | 46.5 | 59.7 | E |
| 7 | -883.9 | -38.8 | 601.1 | 795.2 | -947.1 | -49.5 | -639.3 | -32.5 | D |
| 8 | -658.4 | -85.4 | -312.5 | -94.4 | -660.7 | -95.7 | -612.9 | -86.6 | B |
| 9 | -278.7 | -60.7 | -129.6 | -49.9 | -193.0 | -59.8 | -198.7 | -59.7 | D |
| 10 | NA | NA | NA | NA | NA | NA | NA | NA | E |
| 11 | -291.3 | -91.7 | -142.2 | -83.7 | -228.0 | -90.2 | -223.5 | -88.4 | C |

Histopathologic responders are indicated in gray. All TLG values indicating a percentage difference of >75% were encircled. Abbreviations: ^18^F-FDG PET-CT= Fluorine-18-fluorodeoxyglucose positron emission tomography with computed tomography; SUVmax= maximum standardized uptake value; SUVpeak= peak standardized uptake value; SUVmean= mean standardized uptake value; TLG= total lesion glycolysis; MATV= metabolically active tumor-volume; EORTC-STBSG= European Organization for Research and Treatment of Cancer-Soft Tissue and Bone Sarcoma Group.
